# Appendix Playing Hide and Seek: A Variation to Amyand’s Hernia

**DOI:** 10.7759/cureus.36326

**Published:** 2023-03-18

**Authors:** Ashvind Bawa, Rohin Kansal, Sonalika Sharma, Vinayak Rengan, Pravin Meenashi Sundaram

**Affiliations:** 1 Department of General Surgery, Dayanand Medical College and Hospital, Ludhiana, IND; 2 Department of General Surgery, Dr. Mehta's Hospital, Chennai, IND; 3 Department of General Surgery, Dr. Rengan's Surgical Center, Chennai, IND

**Keywords:** tension free mesh repair, mesh repair, amyand’s hernia, chatgpt, chatgpt improved case report

## Abstract

Amyand's hernia is a rare condition where the appendix becomes trapped in the inguinal hernia sac, leading to severe complications if left untreated. Treatment typically involves surgical repair of the hernia, with the removal of the appendix if necessary. This case report presents a 65-year-old male with compromised cardiac status and a right inguinal hernia, confirmed by ultrasound. The surgery was performed under local anesthesia, and the appendix was normal and reduced back. The patient was discharged on the next day of surgery after an uneventful course in the hospital. There is a difference of opinion regarding the need for an appendectomy in an Amyand's hernia with a normal appendix, with the appendix dancing in and out of the inguinal canal while coughing on the table. The decision to remove or leave a normal appendix in this situation should be based on several factors, including the patient's age, appendix anatomy, and extent of intraoperative inflammation. In conclusion, local anesthesia can be a safe and effective option for patients who are not fit for general or spinal anesthesia. The decision to remove or leave a normal appendix in Amyand's hernia should be based on several factors.

## Introduction

Amyand's hernia is a rare condition that occurs when the appendix becomes trapped in the inguinal hernia sac [1]. If left untreated, it can lead to complications such as appendicitis, abscess formation, sepsis, and even death. The condition was first described by Claudius Amyand, a French surgeon, in 1735 [2]. The occurrence of Amyand's hernia is estimated to be less than 1% of all inguinal hernias [3].

The diagnosis of Amyand's hernia is usually made during surgery, as it is difficult to diagnose based on clinical examination alone. Treatment typically involves surgical repair of the hernia, with the removal of the appendix if necessary [1]. Surgery is the only treatment for this condition, and it is usually performed under general or spinal anesthesia. However, in some cases, patients may not be fit for these types of anesthesia due to underlying medical conditions. This case report discusses the management of a 65-year-old male patient with Amyand's hernia repaired under local anesthesia due to his compromised cardiac condition (primary aortic valve disease).

## Case presentation

A 65-year-old male patient presented to the hospital with a chief complaint of intermittent pain and swelling in the right inguinal region. On examination, there was a swelling in the right inguinal region with a positive cough impulse. The patient was at very high risk for primary aortic valve disease (stage-D) with moderate to severe aortic regurgitation, severe left ventricular and moderate right ventricular systolic dysfunction, moderate pulmonary arterial hypertension, and left ventricular diastolic dysfunction (type-IV). 

Due to the patient's above-mentioned cardiac condition, he was not fit for general or spinal anesthesia, as suggested by the cardiologist and anesthesiologist during his pre-anesthetic check-up. Therefore, the decision was made to perform the surgery under local anesthesia. The patient was counseled regarding the procedure, and informed consent was obtained.

**Figure 1:**
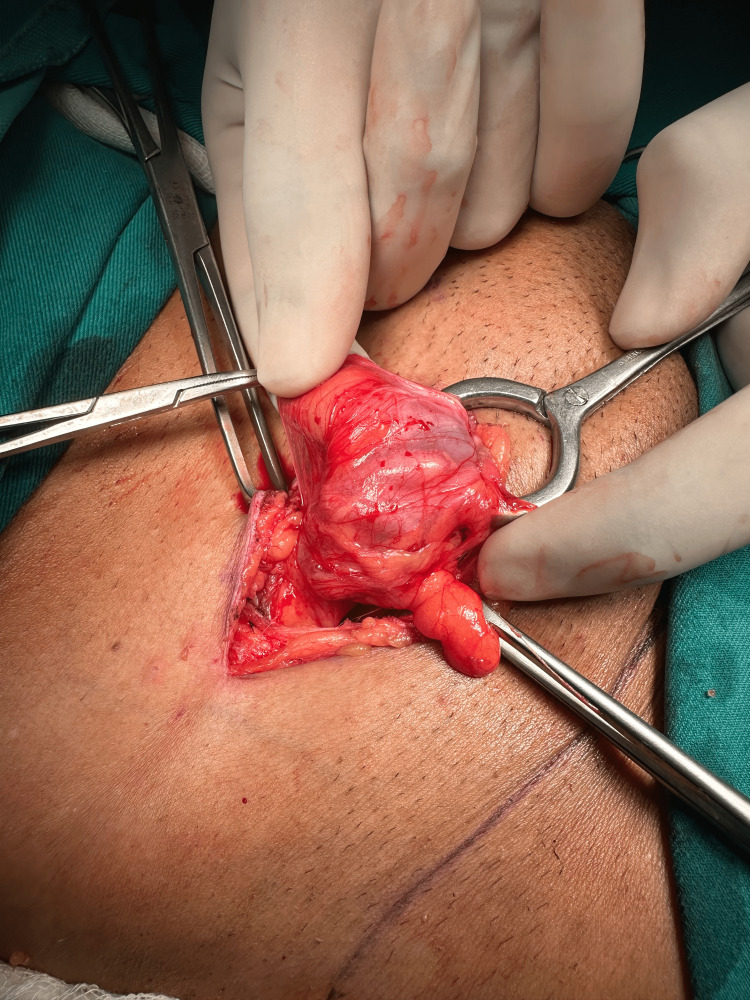
Inguinal Hernia sac with the appendix as its content

The surgical procedure was performed by making a 3 cm incision above and parallel to the inguinal ligament. The hernia sac was identified and dissected, and the appendix could be seen within the indirect sac (Figure 1). On opening the sac, the appendix fell back in. On asking the patient to cough, the appendix kept popping out of the sac and going back spontaneously, as if playing hide and seek (as seen in Video 1). The normal-looking appendix was left alone, and the sac was transfixed and closed. A tension-free hernioplasty was performed using mesh reinforcement, and the incision was closed in layers.

**Video 1:** Protusion of the appendix on cough impulse

The visual analog scale (VAS) was 4 during the surgery, which was managed with additional local anesthesia. Post-operatively, the patient's pain score decreased to 1. The postoperative period was uneventful, and the patient was discharged on the following day after surgery with instructions for wound care and follow-up appointments. The patient was advised to avoid heavy lifting and strenuous activities for the next four weeks. The patient was followed up for two weeks after surgery. The patient did not report any complications, and the surgical site had healed well.

## Discussion

A hernia occurs when a viscus or part of it protrudes through the walls of the containing cavity. The first case of an appendix found in an inguinal hernia was reported by Amyand in 1735, who discovered a perforated appendix in an 11-year-old boy while serving as a sergeant surgeon to King George I and II (2). The occurrence of a normal appendix within the hernia sac ranges from 0.5% to 1%, and left-sided Amyand's hernias have also been reported [4]. The treatment plan should be customized based on the stage of inflammation in the appendix, the presence of abdominal sepsis, and co-morbidities. The Losanoff-Basson classification provides an appropriate guideline for surgical management [5].

The Losanoff-Basson classification offers four types of classification based on the status of the appendix and systemic status, along with surgical management for each type. Type 1 involves a normal appendix and requires hernia reduction with mesh repair. Type 2 involves acute appendicitis without sepsis and requires appendectomy with primary non-mesh repair. Type 3 involves acute appendicitis with peritoneal sepsis and requires laparotomy, appendectomy, and primary non-mesh repair. Type 4 involves acute appendicitis with abdominal pathology and requires management similar to that for types 1-3, with an exploration of any underlying abdominal pathology [5].

Most surgeons agree that synthetic meshes or plugs should not be used in cases of acute appendicitis (Losanoff-Basson types 2-4) within a hernia. However, there is a difference of opinion regarding the need for an appendectomy in cases of type 1 Amyand's hernia with a normal appendix within the inguinal hernia [6]. If an appendix appears normal within a hernia, it may not always need to be removed through an appendectomy. Removing the appendix can introduce the risk of infection to an otherwise sterile procedure. The decision to remove or leave a normal appendix in this situation can be challenging because there is no evidence-based information to guide the decision-making process [7]. The decision should be based on several factors, including the patient's age, the size and anatomy of the appendix, the side of the hernia, and the extent of intraoperative manipulations that may trigger inflammation. Some studies suggest that an appendectomy is unnecessary in Amyand's hernia patients because the condition may remain asymptomatic throughout their lifetime and not require resection [8,9]. During the inguinal hernia repair surgery, we diagnosed the case with Losanoff-Basson type 1, dancing in and out as we made the patient cough. The patient's appendix appeared normal, mobile, and easily reducible back into the abdominal cavity. As the appendix appeared normal, we repositioned it back into the abdominal cavity and closed the defect. We decided not to perform an appendectomy to avoid any complications with mesh placement.

**Figure 2:**
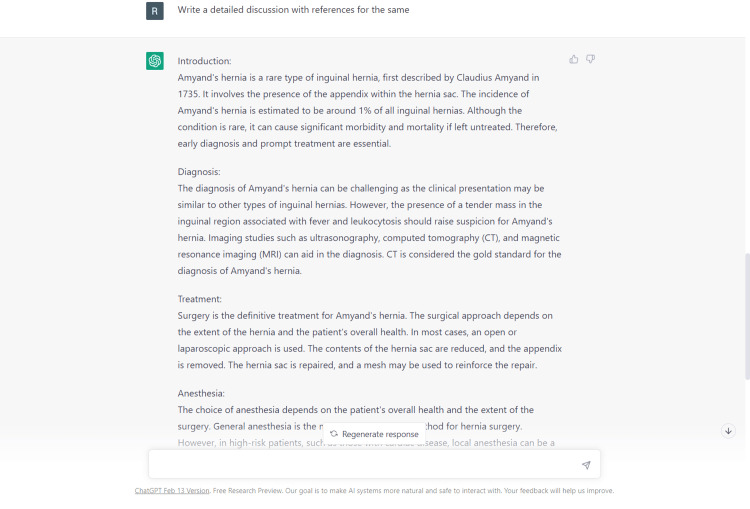
Using ChatGPT for discussion writing

**Figure 3:**
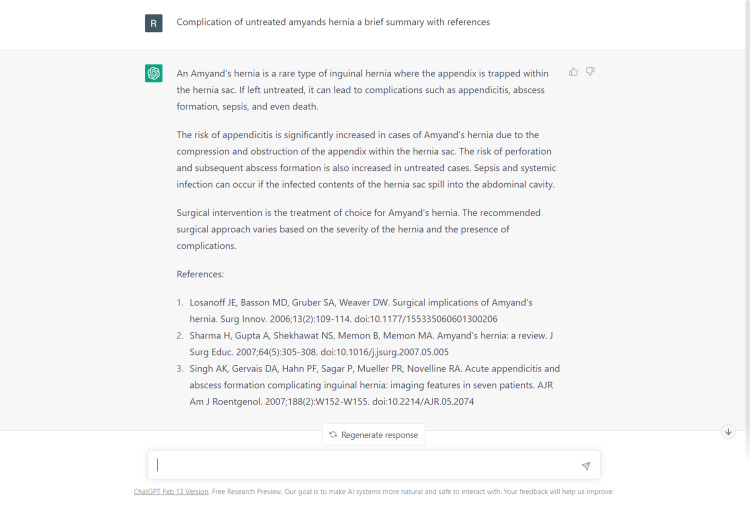
ChatGPT results with references

The case report was drafted using ChatGPT, which proved to be a positive experience for writing the introduction and discussion sections (Figure 2). However, the accuracy of the data provided should be verified for authenticity. Additionally, the references cited did not match the actual data, which can create challenges when using this software for research articles (Figure 3). Despite these drawbacks, ChatGPT is still a useful tool that, if used properly, can be beneficial to some extent for publication purposes.

## Conclusions

Amyand's hernia is an infrequent ailment, and identifying it necessitates a strong suspicion. A CT scan is a valuable method for verifying the diagnosis. The primary form of treatment is surgical intervention. For high-risk patients, local anesthesia surgery can be a safe and effective alternative to general anesthesia in some cases. It is crucial to take the patient's other medical conditions into account when determining the surgical technique and perioperative management. Furthermore, the requirement for appendectomy in Type 1 Amyand's hernia is contentious, and appropriate guidelines are necessary.
